# Serotonin 5-HT_2C_ Receptor Activation Suppresses Binge Intake and the Reinforcing and Motivational Properties of High-Fat Food

**DOI:** 10.3389/fphar.2018.00821

**Published:** 2018-07-27

**Authors:** Amanda E. Price, Noelle C. Anastasio, Sonja J. Stutz, Jonathan D. Hommel, Kathryn A. Cunningham

**Affiliations:** ^1^Center for Addiction Research, University of Texas Medical Branch, Galveston, TX, United States; ^2^Department of Pharmacology and Toxicology, University of Texas Medical Branch, Galveston, TX, United States

**Keywords:** serotonin, 5-HT_2C_ receptor, binge eating, high-fat food, motivation

## Abstract

Binge eating disorder (BED) is characterized by dysfunctional hedonic food intake and reward-related processes. Activation of the serotonin (5-HT) 5-HT_2C_ receptor (5-HT_2C_R) suppresses both food intake and reward-related behaviors and is thus poised to regulate BED. This study assessed the effects of 5-HT_2C_R activation *via* the selective 5-HT_2C_R agonist WAY163909 on binge eating-related behaviors in adult male Sprague-Dawley rats. Low doses of WAY163909 (1.0, 2.0 mg/kg) suppressed high-fat food (HFF) binge intake, but not standard food non-binge intake. WAY163909 (1.0 mg/kg) also attenuated operant responding for self-administered HFF pellets on fixed and progressive ratio schedules of reinforcement, indicating that 5-HT_2C_R activation suppresses the reinforcing and motivational properties of HFF, respectively. These findings suggest that activation of the 5-HT_2C_R may be effective at suppressing binge eating in patients with BED *via* suppression of the reinforcing and motivational properties of HFF. This work supports future studies targeting the 5-HT_2C_R in the treatment of BED.

## Introduction

Binge eating disorder (BED) is characterized by uncontrollable, recurrent episodes of excessive intake of food (American Psychiatric Association, 2013). Of note, food intake during binge episodes is often driven by hedonic rather than homeostatic mechanisms (i.e., food intake driven by wanting and liking factors but not necessary for energy balance; Finlayson et al., 2011; Dalton et al., 2013; Witt and Lowe, 2014). Patients with BED deem high-fat foods (HFFs) more rewarding than people without BED, which may motivate them to consume palatable food (Schebendach et al., 2013), and also exhibit disruptions in reward neurocircuitry (Kessler et al., 2016). Current treatments for BED are comprised of behavioral therapy, off-label use of pharmacotherapies, and lisdexamfetamine, the only clinically approved drug in the treatment of BED (Hutson et al., 2018). One avenue to identify novel treatment approaches in BED is to explore targets known to alter both food intake and reward-related behaviors. The serotonin (5-HT) 5-HT_2C_ receptor (5-HT_2C_R) fulfills these criteria in that previous studies have demonstrated that 5-HT_2C_R activation suppresses feeding behavior (Hewitt et al., 2002; Bickerdike, 2003; Voigt and Fink, 2015) *via* promotion of satiety (for review, Higgins et al., 2017), which in part led to the development and subsequent FDA-approval of the weight loss drug lorcaserin (Belviq^®^), a first-in-class selective 5-HT_2C_R agonist (Fidler et al., 2011; O’Neil et al., 2012; Nigro et al., 2013; Aronne et al., 2014). In addition, preclinical studies demonstrate that 5-HT_2C_R activation also regulates the hedonic properties of rewarding substances such as food and drugs of abuse (for reviews, Higgins and Fletcher, 2003, 2015; Fletcher et al., 2010; Cunningham and Anastasio, 2014). Thus, suppression of the reinforcing and motivational properties of palatable food *via* 5-HT_2C_R activation may be one mechanism to decrease hedonic eating and ultimately binge intake.

The investigational compound WAY163909 is a high affinity, full efficacy 5-HT_2C_R agonist relative to the homologous 5-HT_2A_R and 5-HT_2B_R (Dunlop et al., 2005). WAY163909 suppresses food intake in Sprague-Dawley rats, obese Zucker rats, and diet-induced obese mice, effects that are completely reversed by a selective 5-HT_2C_R antagonist (Dunlop et al., 2005). Furthermore, WAY163909 decreases intake of both drug and sucrose reinforcers (Cunningham et al., 2011; Anastasio et al., 2014; Swinford-Jackson et al., 2016; Berro et al., 2017). To our knowledge, WAY163909 has not been previously tested for efficacy in preclinical binge eating models. Herein, we tested the hypothesis that WAY163909 would suppress binge intake of HFF in an intermittent access model at doses that do not interrupt standard food (SF) non-binge intake in adult male Sprague-Dawley rats. We further explored if WAY163909 alters hedonic eating *via* suppression of the reinforcing and motivational properties of HFF in two operant conditioning paradigms. These studies provide valuable insight into the potential therapeutic applicability of 5-HT_2C_R activation in BED.

## Materials and Methods

### Animals

Naïve male, outbred Sprague-Dawley rats (*n* = 42; Harlan, Houston, TX, United States) weighing 200–225 g at arrival were housed two per cage (except where noted below) under a 12-h light-dark cycle (lights on between 0600 and 1800 hours) with controlled temperature (21–23°C) and humidity (40–50%). Animals were acclimated for 7 days to the colony room prior to handling and experimental procedures. SF and water were available to rats *ad libitum* except during daily operant sessions and where noted below. All experiments were conducted in accordance with the NIH *Guide for the Care and Use of Laboratory Animals* (2011) and with the University of Texas Medical Branch Institutional Animal Care and Use Committee approval.

### Food

Standard food available *ad libitum* and used in non-binge intake studies consisted of 25% protein, 58% carbohydrate, and 17% fat (by kcal; Teklad LM-485 Mouse/Rat Sterilizable Diet; Teklad Diets, Madison, WI, United States; 3.1 kcal/g). HFF employed for binge intake studies contained 20% protein, 35% carbohydrate, and 45% fat (by kcal; D12451, Research Diets, New Brunswick, NJ, United States; 4.73 kcal/g). HFF pellets used in operant assays consisted of 16% protein, 38% carbohydrate, and 46% fat (by kcal; BioServ product #F06162, 45 mg/pellet, Flemington, NJ, United States; 4.60 kcal/g).

### Drugs

WAY163909 [(7b-R,10a-R)-1,2,3,4,8,9,10,10a-octahydro-7bH-cyclopenta[b][1,4] diazepino [6,7,1hi]indole] was a gift from Pfizer, Inc. (New York, NY, United States) and was dissolved in 0.9% NaCl (vehicle, VEH). WAY163909 was tested at a dose range (0–2 mg/kg) that did not alter total horizontal ambulation in a motor activity monitor (3 mg/kg WAY163909 has been shown to significantly suppress total horizontal ambulation) but dose-dependently suppressed operant responding for self-administered sucrose pellets (Cunningham et al., 2011). These effects are completely blocked following pretreatment with the selective 5-HT_2C_R antagonist SB242084 (Dunlop et al., 2005; Cunningham et al., 2011). All injections were administered intraperitoneally (i.p.) in a volume of 1 ml/kg.

### HFF Binge Intake

Binge intake of HFF was assessed as described previously in a HFF intermittent access model (Benzon et al., 2014). Briefly, single-housed rats (*n* = 9) were acclimated to exclusive *ad libitum* access to HFF for 1 week to prevent food neophobia. Following this acclimation timeframe, rats were provided with exclusive *ad libitum* access to SF except during binge intake testing. The effects of 5-HT_2C_R activation on binge intake were determined after i.p. injection of vehicle or 0.5, 1, or 2 mg/kg WAY163909 15 min prior to the beginning of the dark cycle (1745 hours). At 1800 hours, SF was removed and 40 g of HFF was added to the home cage. HFF was removed 2 h later (2000 hours) and weighed to determine binge intake. Rats were then provided *ad libitum* access to SF. All rats received each of the four pharmacological treatments prior to HFF binge intake testing in a randomized manner with testing spaced at least 1 week apart. Previously published results using this paradigm demonstrate that rats receiving continuous access to HFF eat an average of 5 g of HFF in 2 h, whereas rats subjected to intermittent access to HFF eat an average of 7 g of HFF in 2 h (i.e., binge intake; Benzon et al., 2014). Thus, rats which consumed <5 g HFF in 2 h after vehicle pretreatment were excluded for not exhibiting HFF binge intake (*n* = 2). An additional rat was excluded as an outlier (i.e., intake greater than two standard deviations from the mean).

### SF Non-binge Intake

The effect of 5-HT_2C_R activation on non-binge SF intake was also assessed in single-housed rats (*n* = 8) that were injected i.p. with vehicle or 0.5, 1, or 2 mg/kg WAY163909 15 min prior to the beginning of the dark cycle (1745 hours). At 1800 hours, all but 40 g of SF was removed from the home cage. At the end of 2 h (2000 hours), SF was removed and weighed to determine non-binge intake, and rats were allowed *ad libitum* access to SF. Rats received each of the four treatments prior to non-binge intake testing in a randomized manner at least 1 week apart.

### Operant Conditioning for Self-Administration of HFF Pellets

Rats were trained to self-administer HFF pellets *via* an operant conditioning paradigm. Operant studies took place between 0900 and 1200 hours in standard operant chambers housed within a ventilated and sound-attenuated chamber and equipped with two retractable levers (Med Associates, Georgia, VT, United States). Operant studies consisted of 30-min sessions (5 days/week) during which rats were trained to lever press for a HFF pellet. Completion of the fixed ratio (FR) or progressive ratio (PR) schedule of reinforcement on the active lever resulted in delivery of the reinforcer (one HFF pellet); on the FR schedule, pellet delivery was paired with a discrete, flashing light. There were no scheduled consequences for lever presses on the inactive lever.

#### Effects of WAY163909 on FR Responding

Rats (*n* = 9) were SF restricted to 85–90% of free-feeding levels for the first 3 days of operant conditioning to facilitate acquisition of HFF self-administration and then provided with *ad libitum* access to SF while in the home cage for the remainder of the study. Rats were trained on an FR1 schedule of reinforcement for HFF pellets for 5 days, an FR3 schedule of reinforcement for 2 days, and moved to an FR5 schedule of reinforcement for the remainder of the study. The criterion for stable FR acquisition and responding (<25% variability in the number of HFF pellets earned over three consecutive FR5 training sessions) was achieved prior to initiation of test sessions. Once stable, rats underwent two consecutive days of testing in which they received vehicle on 1 day and 1 mg/kg WAY163909 the following day. After allowing at least 3 days for washout of drug and re-establishment of stability (Cunningham et al., 2011), rats underwent two additional consecutive days of testing in which they received vehicle on 1 day and 0.3 mg/kg WAY163909 the following day. All injections were administered i.p. 15 min prior to the beginning of the operant session. One rat was removed from the study for failure to achieve the stability criterion.

#### Effects of WAY163909 on PR Responding

Rats (*n* = 16) were SF restricted to 85–90% of free-feeding levels for the first 3 days of operant conditioning to facilitate acquisition of HFF self-administration and then provided with *ad libitum* access to SF while in the home cage for the remainder of the study. Rats were trained on an FR1 schedule of reinforcement for HFF pellets for 5 days, an FR3 schedule of reinforcement for 2 days, an FR5 schedule of reinforcement for 2 days, and then moved to a PR schedule of reinforcement for the remainder of the study. The PR schedule of reinforcement (1, 2, 4, 6, 9, 12, 15, 20, 25, 32, 40, 50, 62, 77, and 95) required rats to progressively increase the number of active lever presses needed to receive a single HFF reinforcer (Richardson and Roberts, 1996; McCue et al., 2017). PR sessions ended 10 min after the last reinforcer was received. The criterion for stable PR acquisition and responding (<25% variability in the number of HFF pellets earned over three consecutive PR training sessions) was achieved prior to initiation of test sessions. Following achievement of stability, rats were tested with 1 mg/kg of WAY163909, 0.3 mg/kg of WAY163909, and vehicle with at least 3 days between tests to allow for washout of drug and re-establishment of stability (Cunningham et al., 2011). All injections were administered i.p. 15 min prior to the beginning of the operant session. Four rats were excluded for not achieving stability and an additional rat was removed from the study due to an equipment malfunction.

### Statistical Analyses

A repeated measures, one-way analysis of variance (ANOVA) was employed to assess the main effect of WAY163909 treatment on HFF binge intake, 2-h SF non-binge intake, and measures of operant responding for self-administration of HFF pellets (i.e., active and inactive lever presses, pellets earned, breakpoint, and latency to first reinforcer; Cunningham et al., 2011). Subsequent *a priori* comparisons to vehicle were analyzed using a one-tailed Dunnett’s procedure. A paired Student’s *t*-test was employed to assure consistent baseline responding between FR vehicle test days and between the days preceding the first and last PR tests. All statistical analyses were conducted with an experiment-wise error rate of α = 0.05 in SAS for Windows 9.4. Power analyses were completed using SPSS Statistics Version 25.

## Results

### HFF Binge and SF Non-binge Intake

**FIGURE 1:**
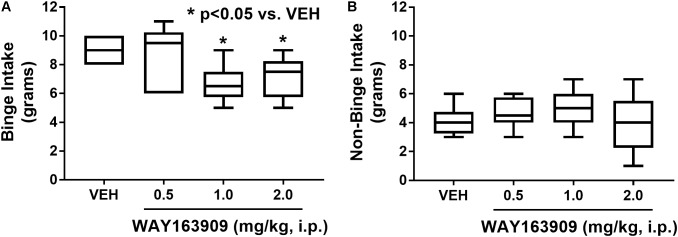
WAY163909 preferentially suppresses high-fat food (HFF) binge intake over standard food (SF) non-binge intake. **(A)** WAY163909 (1.0, 2.0 mg/kg) suppressed HFF binge intake in an intermittent access binge intake paradigm (*n* = 6). **(B)** WAY163909 did not alter SF non-binge intake (*n* = 8). ^∗^*p* < 0.05 vs. VEH (Dunnett’s test); boxes extend from the 25th to 75th percentiles, with whiskers representing minimum and maximum values and the line within the box representing the median value.

The effect of the 5-HT_2C_R agonist WAY163909 on HFF binge intake was assessed in an intermittent HFF access model (*n* = 6). A repeated measures, one-way ANOVA revealed a main effect of treatment (*F*_3,15_ = 4.79; *p* = 0.0155). *A priori* comparisons demonstrated that 1 and 2 mg/kg of WAY163909 significantly suppressed binge intake compared to vehicle treatment (*p* < 0.05; **Figure 1A**). A power analysis determined an achieved power of 0.806 for this assessment. These data indicate that activation of 5-HT_2C_R signaling suppresses binge eating. The effect of WAY163909 on 2-h, non-binge SF intake was also assessed (*n* = 8). A repeated measures one-way ANOVA revealed no main effect of treatment (*F*_3,21_ = 1.12; *p* = 0.3642; **Figure 1B**). These results are consistent with published results indicating the ED_50_ for WAY163909-induced suppression of 2-h food intake in 24-h fasted Sprague-Dawley rats is 2.93 mg/kg (Dunlop et al., 2005). Together, these data indicate that lower doses of WAY163909 preferentially suppress HFF binge intake over non-binge intake of SF.

### Operant Responding for Self-Administration of HFF Pellets

**FIGURE 2:**
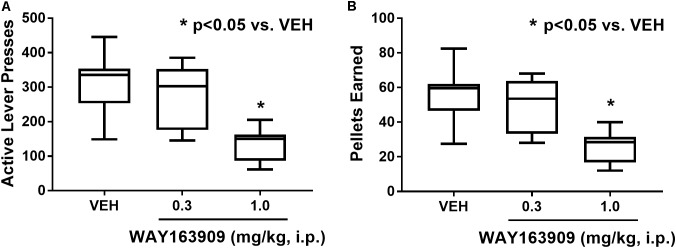
WAY163909 suppresses the reinforcing efficacy of high-fat food (HFF). WAY163909 (1.0 mg/kg) suppressed **(A)** active lever presses and **(B)** pellets earned during self-administration of HFF pellets on a fixed ratio schedule of reinforcement (*n* = 8). ^∗^*p* < 0.05 vs. VEH (Dunnett’s test); boxes extend from the 25th to 75th percentiles, with whiskers representing minimum and maximum values and the line within the box representing the median value.

The effect of WAY163909 on the reinforcing value of HFF was assessed using FR responding for HFF pellets in freely fed rats (*n* = 8). A paired Student’s *t*-test between the first and second vehicle test indicated there were no significant differences in active lever presses (*p* = 0.934), inactive lever presses (*p* = 0.621), pellets earned (*p* = 0.605), or latency to first reinforcer (*p* = 0.089); thus, the average vehicle response was used as control for analysis of the WAY163909 dose–response relationship. A repeated measures, one-way ANOVA revealed a main effect of treatment on active lever presses (*F*_2,14_ = 47.54; *p* < 0.0001) and pellets earned (*F*_2,14_ = 42.08; *p* < 0.0001), but not for inactive lever presses (*F*_2,14_ = 0.34; *p* = 0.7185) or latency to first reinforcer (*F*_2,14_ = 1.39; *p* = 0.2819). *A priori* comparisons indicated that 1 mg/kg of WAY163909 significantly suppressed active lever presses (*p* < 0.05) and pellets earned (*p* < 0.05) compared to vehicle (**Figure 2**). These data indicate that activation of the 5-HT_2C_R suppresses the reinforcing value of HFF, a finding which coalesces with previously published results demonstrating that WAY163909 also dose-dependently suppresses operant responding for self-administration of sucrose pellets (Cunningham et al., 2011).

**FIGURE 3:**
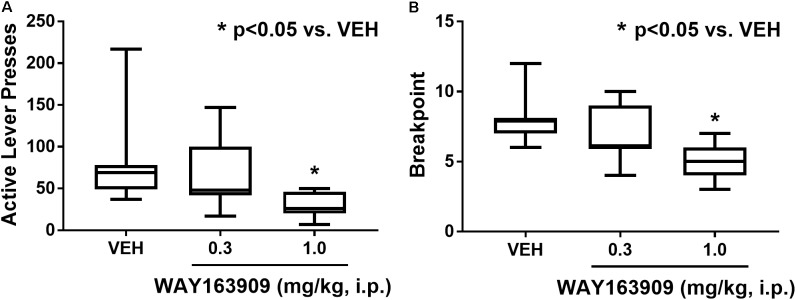
WAY163909 suppresses the motivational value of high-fat food (HFF). WAY163909 (1.0 mg/kg) suppressed **(A)** active lever presses and **(B)** breakpoint during self-administration of HFF pellets on a progressive ratio schedule of reinforcement (*n* = 11). ^∗^*p* < 0.05 vs. VEH (Dunnett’s test); boxes extend from the 25th to 75th percentiles, with whiskers representing minimum and maximum values and the line within the box representing the median value.

The effect of WAY163909 on the motivational value of HFF was assessed using PR responding for HFF pellets in freely fed rats (*n* = 11). A paired Student’s *t*-test was used to assess consistent baseline responding between the days preceding the first and last test sessions; analyses indicated there was no difference in active lever presses (*p* = 0.819) or pellets earned (*p* = 0.714). A repeated measures one-way ANOVA revealed a main effect of treatment on active lever presses (*F*_2,20_ = 9.12; *p* = 0.0015) and breakpoint (*F*_2,20_ = 19.11; *p* < 0.0001), but not inactive lever presses (*F*_2,20_ = 2.59; *p* = 0.0998) or latency to first reinforcer (*F*_2,20_ = 1.81; *p* = 0.1888). *A priori* comparisons indicated that 1 mg/kg of WAY163909 significantly suppressed active lever presses (*p* < 0.05) and breakpoint (*p* < 0.05) compared to vehicle treatment (**Figure 3**). These data indicate that 5-HT_2C_R activation suppresses the motivational value of HFF.

## Discussion

Food intake can be described as homeostatic (intake necessary to maintain energy balance) or hedonic (intake driven by reward-related factors; Lutter and Nestler, 2009). Hedonic processes are postulated as an important component of binge eating episodes seen in disorders such as BED (Finlayson et al., 2011). To model binge eating in rodents, we gave rats *ad libitum* access to standard chow along with intermittent access to a highly palatable food (Corwin and Wojnicki, 2006; Corwin et al., 2011; Benzon et al., 2014). The palatable food used in this study was a HFF chow, which is nutritionally representative of foods that patients with BED may eat in excess during a binge episode (Corwin et al., 2011). Administration of 1 mg/kg of WAY163909 significantly suppressed HFF binge intake in rodents, suggesting that 5-HT_2C_R activation may be a viable therapeutic approach to suppress binge eating episodes in patients with BED. This same dose of WAY163909 did not alter non-binge intake, consistent with previous literature that higher doses of WAY163909 (i.e., 3–10 mg/kg) are required to suppress 2-h SF intake in 24-h fasted male Sprague-Dawley rats (Dunlop et al., 2005). This suggests that 5-HT_2C_R activation preferentially suppresses hedonic intake of food at lower doses but attenuates both hedonic and homeostatic intake of food at higher doses. We then demonstrated that 1 mg/kg of WAY163909 suppressed both FR and PR responding, suggesting that 5-HT_2C_R activation attenuates the reinforcing efficacy and motivational properties of HFF, respectively. These findings are congruent with literature demonstrating 5-HT_2C_R activation decreases intake of other types of palatable food, such as those high in carbohydrates (Martin et al., 1998; Fletcher et al., 2010; Higgins et al., 2013b).

Activation of the 5-HT_2C_R is postulated to primarily suppress feeding by increasing production of α-melanocyte stimulating hormone which acts on melanocortin 4 receptors in the paraventricular nucleus of the hypothalamus to promote satiety (Heisler et al., 2002; Xu et al., 2008; Lam et al., 2008). However, 5-HT_2C_R activation-induced suppression of operant responding for food in self-administration studies suggests that the 5-HT_2C_R regulates intake of food in additional ways since total food intake in these paradigms is often not enough to promote satiety (Higgins et al., 2013a). Previous studies have also indicated that 5-HT_2C_R agonists suppress intake of palatable food in non-food deprived rats (Rowland et al., 2008; Canal et al., 2014). A recent study demonstrated that the clinically approved 5-HT_2C_R agonist lorcaserin suppressed both binge-like eating and hunger-driven feeding in wild-type mice (Xu et al., 2017). Interestingly, lorcaserin-induced suppression of binge-like eating in mice is dependent upon 5-HT_2C_R expression on dopaminergic neurons in the ventral tegmental area, cells highly implicated in reward-related behaviors (Xu et al., 2017). In mice with selective knockout of 5-HT_2C_R in dopaminergic neurons, lorcaserin is unable to suppress binge-like eating, suggesting that the 5-HT_2C_R is mediating its effects on hedonic feeding through mechanisms beyond hypothalamus-dependent promotion of satiety (Xu et al., 2017). Indeed, lorcaserin also suppresses PR responding for chocolate pellets *via* activation of 5-HT_2C_R in the ventral tegmental area (Valencia-Torres et al., 2017), further supporting a role for mesolimbic 5-HT_2C_R in the control of palatable food intake. To our knowledge, the role of the 5-HT_2C_R in hypothalamic neurons has not been explored in binge eating behavior, but future studies should assess if the 5-HT_2C_R in hypothalamic subregions contributes to WAY163909-mediated suppression of binge eating because of the well-established role of this population in mediating food intake.

Elevations in body weight are seen in approximately 70% of patients with BED (Kessler et al., 2013). Thus, a medication that effectively suppresses both binge episodes and overall food intake at therapeutic doses would be highly beneficial in the treatment of comorbid BED and obesity. The FDA-approved selective 5-HT_2C_R agonist lorcaserin may be of benefit for this population of patients. Interestingly, clinical trials for lorcaserin showed that while, on average, treatment produced modest effects (about 3% weight loss when accounting for the effects of placebo), lorcaserin treatment resulted in 5% or even 10% body weight loss in certain subpopulations, a phenomenon occurring at twice the frequency in the lorcaserin group compared to the placebo group (Aronne et al., 2014). Our findings combined with previous literature suggest that 5-HT_2C_R activation suppresses hedonic intake of food in addition to homeostatic intake of food; thus, lorcaserin may show higher efficacy in patients seeking weight loss treatment with comorbid BED compared to patients without BED. To our knowledge, a behavioral subtyping of the individuals who exhibit the greatest weight loss upon lorcaserin treatment has not yet been reported. The present study suggests that analyses assessing the efficacy of lorcaserin in different behavioral subtypes of obesity are warranted.

This study supports 5-HT_2C_R activation as a novel therapeutic target to suppress hedonic food intake in patients with BED. Future studies should assess the viability of repurposing the weight loss drug, lorcaserin, in the treatment of BED. These findings, in combination with the larger body of literature surrounding the role of 5-HT_2C_R in food intake, suggest that 5-HT_2C_R activation may be especially helpful in the treatment of comorbid BED and obesity.

## Author Contributions

NA, JH, and KC planned the experiments. NA and SS completed the experiments. AP and NA analyzed the data and interpreted the results. AP drafted the manuscript. All authors edited and approved the manuscript.

## Conflict of Interest Statement

The authors declare that the research was conducted in the absence of any commercial or financial relationships that could be construed as a potential conflict of interest.
